# Analysis of Biogenic Amines Using Immunoassays, HPLC, and a Newly Developed IC-MS/MS Technique in Fish Products—A Comparative Study

**DOI:** 10.3390/molecules26206156

**Published:** 2021-10-12

**Authors:** Drago Kočar, Sevim Köse, Serkan Koral, Bekir Tufan, Andrej Ščavničar, Matevž Pompe

**Affiliations:** 1 Faculty of Chemistry and Chemical Technology, University of Ljubljana, Večna pot 113, 1000 Ljubljana, Slovenia; andrej.scavnicar@gmail.com (A.Š.); matevz.pompe@fkkt.uni-lj.si (M.P.); 2 Faculty of Marine Sciences, Karadeniz Technical University, Çamburnu, 61530 Trabzon, Turkey; kosesevim@gmail.com (S.K.); bekirtufan@gmail.com (B.T.); 3 Faculty of Fisheries, İzmir Katip Çelebi University, 35640 İzmir, Turkey; serkan.koral@ikcu.edu.tr

**Keywords:** biogenic amines, IC-MS/MS, HPLC, immunoassays, method comparison, fish products, histamine

## Abstract

In this study, comparative analyses were carried out with ion chromatography mass-spectrometry (IC-MS/MS) which has no derivatization step, high-performance liquid chromatography (HPLC) technique, as well as two quantitative and two semi-quantitative immunoassays. The results demonstrated that HPLC and quantitative immunoassay methods were well-correlated with IC-MS/MS in determining histamine in various types of fish products. The best correlation was observed with the HistaSure ELISA Fast Track kit (R^2^ = 0.9903). More than half of the values (68%) obtained by two methods were also statistically similar. The results of semi-quantitative test kits also supported histamine values estimated by quantitative methods, with some exceptions. The best results were found for HistaSure Lateral Flow in supporting the quantitative techniques. Therefore, these methods are found suitable for monitoring histamine in fish products in terms of food safety. Good correlations were also observed HPLC and IC-MS/MS in determining cadaverine, putrescine, and tyramine with the highest value observed for tyramine as R^2^ = 0.9785. However, no correlation was observed for other biogenic amines, and the majority of the results were significantly different from each other for these amines (*p* < 0.05). The differences may be caused by the drawbacks reported previously for HPLC. However, further studies are required to confirm the possible effects. This study provides a comparative evaluation of several methods in terms of their suitability in determining biogenic amines in fish products for both monitoring and regulatory purposes.

## 1. Introduction

Biogenic amines (BAs) are a group of biologically active compounds synthesized from free amino acids mainly as a consequence of decarboxylation activity and are commonly found in foods and animal feeds. Their occurrence in foodstuffs is abundant especially in fish and meat products mainly as a result of bacterial growth and spoilage. Foodborne BAs are considered to be potential toxins and are often produced by spoilage microorganisms [1,2].

Histamine is the most commonly investigated BA due to its association with histamine food poisoning (HFP), previously known as “scombroid fish poisoning” due to its first involvement being reported from *Scombridae* family such as bonito and tuna. Although a level of 1 g histamine/kg foods was considered necessary to induce a toxic response in humans, lower amounts down to 50 mg/kg were also reported to cause HFP. In most cases, histamine levels with implicated fish have been above 200 mg/kg, often above 500 mg/kg [2,3]. Therefore, the levels of histamine in fish and fish products are regulated by various food and government authorities of most countries [2,4,5,6,7]. Since histamine is produced from precursor histidine, the levels of this amino acid which varies to great extend in foods are crucial in the regulations. Therefore, the European Union (EU) Directive [5,6] set legal limits for six fish families (namely, *Engraulidae*, *Clupeidae*, *Coryfenidae*, *Pomatomidae*, *Scombridae*, and *Scombresosidae*) for histamine levels. However, various other fish species and foods have been reported to contain high histidine levels [2,7,8]. According to EU regulation, nine samples must be taken from each batch of fishery products belonging to these families and these samples must fulfill the following requirements.

The average histamine content must be 100 mg/kg or less, while no more than two samples may have levels between 100 mg/kg and 200 mg/kg, and no sample may have a level above 200 mg/kg. However, higher amounts are allowed for “the fishery products” that have undergone enzyme maturation treatment in brine as the average histamine content must be 200 mg/kg or less, no more than two samples may have levels between 200 mg/kg and 400 mg/kg, and no sample may have a level above 400 mg/kg. For fish sauce, only one sample is to be taken, and histamine content should not exceed 400 mg/kg [5,6]. The Food and Drug Administration of USA (FDA) [9] set a stricter upper allowable limit for histamine as 50 mg/kg for fish species, since histamine is generally not uniformly distributed in decomposed fish, and numerous outbreaks were caused by this amount. Other countries in the world usually follow the rules of either the EU or FDA although some differences are existing that can be obtained in detail in the recent review of DeBeer et al. [10].

Although no regulations were made for other BAs in fish products, the toxicity of these amines has been reported. Putrescine and cadaverine play an important role in food poisoning as they can potentiate histamine toxicity [11] while phenylethylamine and tyramine are reported to be toxic to susceptible individuals [4]. For adults, values of 100–800 mg/kg for dietary tyramine and 30 mg/kg for dietary phenylethylamine have been reported as toxic doses. The ingestion of 60 mg/kg of dietary tyramine can cause migraines in individuals using monoamine oxidase inhibitor drugs, while 100–250 mg/kg will produce a hypertensive crisis [12]. Moreover, several BAs have been reported to cause carcinogenic nitrosamines. Furthermore, BAs have been also used as chemical indicators of seafood quality due to their presence in food changes during processing and storage [2,13]. Therefore, although none of these amines other than histamine are regulated either for fish products or other food products, analysis of BAs is important in terms of maintaining food safety and also determining seafood quality during processing, storage and marketing.

A great number of methods so far have been employed to analyze BAs in food, particularly fish [2,14,15,16,17,18,19]. Quantitative methods such as chromatographic techniques are generally used for both regulatory and monitoring purposes while qualitative methods such as enzyme immunoassays are only used for a rapid screening test for histamine only to be applied in the seafood companies’ hazard analysis critical control point (HACCP) systems for monitoring and prevention purposes. These methods have been compared by taking the factors of sensitivity, linearity, rapidity, repeatability, efficiency, and operational requirements into consideration, and advantages and disadvantages have been discussed by various authors over the years [2,15,16,17,18,19,20,21,22,23,24,25,26,27]. For regulatory purposes, reference methods are preferred by the government authorities. These methods are also critical to support the development of new methods, to verify the results of rapid screening, and for settling trade disputes [8]. So far, two reference methods have been approved. One of which is the batch fluorescence method using conventional fluorescence spectrometry and ion exchange clean-up (AOAC 977.13) recognized as the reference method of Codex Alimentarius [8]. European Commission has approved the High-Performance Liquid Chromatography (HPLC) method developed by Duflos et al. [28] based on pre-column derivatization with dansyl chloride and internal standard 1,3-diaminopropane [5,28]. This method was modified by changing the internal standard to 1,7-diaminoheptane and an inter-laboratory study was initiated [8,28]. Their study revealed sample matrix issues leading to poor recoveries for some matrices. Stroka et al. [25] compared the modified version of the EC-approved HPLC method against the Codex approved fluorescence method (AOAC 977.13) and again the same matrix issues were encountered [8,25].

An interest in ”portable” procedures for field analysis capable of rapid screening fishery products dockside has led to the development of commercial test kits proposed for HACCP plan applications [20]. Commercial test kits based on immunoassay methods for histamine analyses became popular because of their user-friendliness and reduced time requirements compared to those of traditional analytical techniques. However, they are easily affected by the sample matrix and other application conditions. Therefore, it is important that their suitability for the analysis of histamine in various fisheries products has to be evaluated against a reliable quantitative method. For this purpose, a few studies have been carried out for different types of test kits to be evaluated for different foods, particularly fish and fish products using various quantitative methods [20,21,23,29]. Among these studies, EC-approved HPLC and Codex-approved fluorescence method (AOAC 977.13) were commonly used as reference methods for the evaluation of test kits for fish and fish products [20,30,31,32]. The results of previous studies indicated that the performance of test kits can vary according to the type of product and the method used [21,23].

Önal [14] emphasized that one of the highest drawbacks in the analysis of BAs in food is the complexity of the sample matrix. Because of the presence of potentially interfering compounds in the sample matrix, the several BAs simultaneously cannot be determined in the analysis. Moreover, the necessity of derivatization of BAs in most analytical procedures such as HPLC was reported to lead to further interfering compounds. Therefore, the analysis of BAs without derivatization has been a current interest amongst food analysts. Such application has been mostly carried out by using liquid chromatography-mass spectrometric (LC-MS) detections. So far, a few methods have been developed for the analysis of BAs in different foods such as meat, cheese and wine [33,34,35,36,37]. Recently, we have developed an ion chromatography-mass spectrometric detection (IC-MS/MS) method for analyzing BAs in cheese, fish and fisheries products which applies the direct application of the sample extract for reading BAs in these products without derivatization [26,27].

The earlier studies demonstrated that the IC-MS/MS method showed a good recovery for various BAs in cheese and fish products. The method showed high reducibility and sensitivity, and low detection limits (with the exception of spermidine and spermine) in determining BAs in fish and cheese [26,27]. So far, few quantitative test kits have been validated by the AOAC Research Institute Performance Tested Method SM program, namely, HistaSure™ ELISA (Fast Track) also called Histamine FOOD EIA (by Labor Diagnostika Nord, Nordhorn, Germany, (#021402) and enzyme immunoassay (EIA) (by Kikkoman Biochemifa Company, Tokyo, Japan) Certificate (#042802), and the BioSystems^®^ Y15 Histamine Dehydrogenase kit (#072001) (by BioSystems S.A., Barcelona, Spain) [29,31,38]. Manz and Booltink [32] validated their immunoassay test kit for histamine analysis called HistaSure™ ELISA (Fast Track) against Codex-approved fluorescence method (AOAC 977.13) and obtained good correlation while Köse et al. [23] compared various quantitative and qualitative immunoassay test kits to EC-approved HPLC method in estimating histamine in fish products with varying recoveries and performances. However, no study exists on the compatibility of commercial quantitative immunoassay histamine methods against IC-MS/MS method which has the advantage of eliminating the drawbacks obtained by the derivatization steps of the EU approved HPLC and Codex-approved fluorometric method. Therefore, it is important to compare this method against qualitative and quantitative immunoassays. Our earlier study with various commercial immunoassay test kits showed that few test kits were compatible with the EU-approved HPLC method for estimating histamine in traditional fish products [23]. However, there is no study compared to the compatibility of commercial histamine test kits neither with LC-MS/MS or our currently developed IC-MS/MS which has no derivatization step that may lead to matrix effect or poor recoveries. Therefore, there is still a need to evaluate recently developed IC-MS/MS method against some commercial test kits as well an EC-approved HPLC method in terms of analyzing biogenic amines for monitoring and regulatory purposes.

Therefore, this study aimed to evaluate the performance of different quantitative and semi-quantitative immunoassay test kits, EC-approved HPLC method against recently developed IC-MS/MS method in determining BAs in various fish and fish products which have different sample matrixes. Since the quantitative immunoassay HistaSure™ ELISA (Fast Track) was not evaluated against EC-approved HPLC for the analysis of BAs in fish, it was included in this study along with Histamine Food ELISA which was previously evaluated against HPLC, respectively. Moreover, two semi-quantitative immunoassay test kits from the same company were included in the study in order to compare their performance against currently developed IC-MS/MS in the presence of various matrixes of fish products.

## 2. Results and Discussion

Table 1 shows the histamine values in 32 fish samples obtained by the methods of the IC-MS/MS, HPLC, and four test kits. Histamine values in samples varied from below detection limits and up to around 320 mg/kg. Seven samples were not analyzed by the IC-MS/MS method due to problems that occurred during analysis, sample storage and transportation. Moreover, histamine value of one sample (No. 18) was out of range of normal distribution. Therefore, the histamine values of 24 samples were used in the correlation tests.

**Figure 1:**
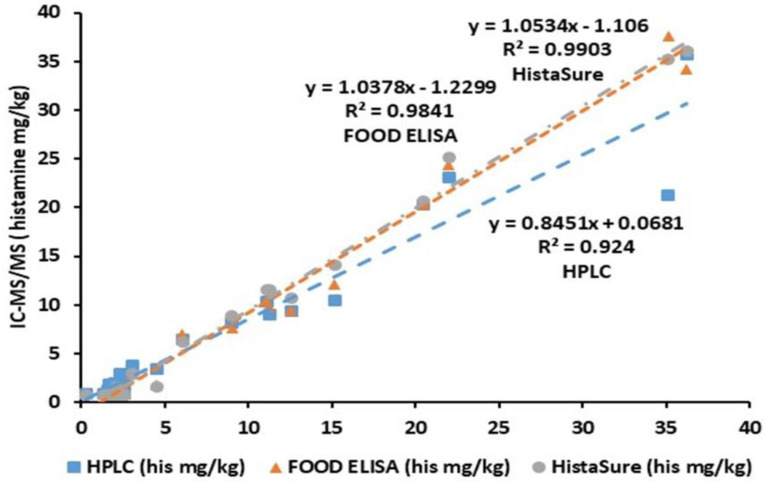
Correlation results of three quantitative methods against IC-MS/MS in determining histamine in various fish products.

Correlation analysis demonstrated that all the methods had a good correlation with the IC-MS/MS method (Figure 1). The best correlation was found with the HistaSure ELISA test kit (Fast Track) (R^2^ = 0.9903) while the worst correlation was obtained with the HPLC method (R^2^ = 0.924). Different superscript lowercase letters in the same row in Table 1 represent statistical differences amongst the methods (*p* < 0.05). According to these results, overall 68% of the histamine values of the fish samples analyzed by HistaSure ELISA, which is AOAC approved method were found significantly similar to the IC-MS/MS, indicating compatibility of this test kit (*p* > 0.05). Most disagreements among the results usually occurred for the low levels of histamine, particularly below detection/quantification limits. The histamine values obtained by the HPLC method took the second place in comparison with IC-MS/MS and 56% of the results were significantly similar to each other (*p* > 0.05). Only about half of the histamine results (48%) obtained by FOOD ELISA method were statistically similar to the values obtained by the IC-MS/MS (*p* > 0.05). One of the marked differences corresponded to a sample named salmon *Gravadlax*.

**Figure 2:**
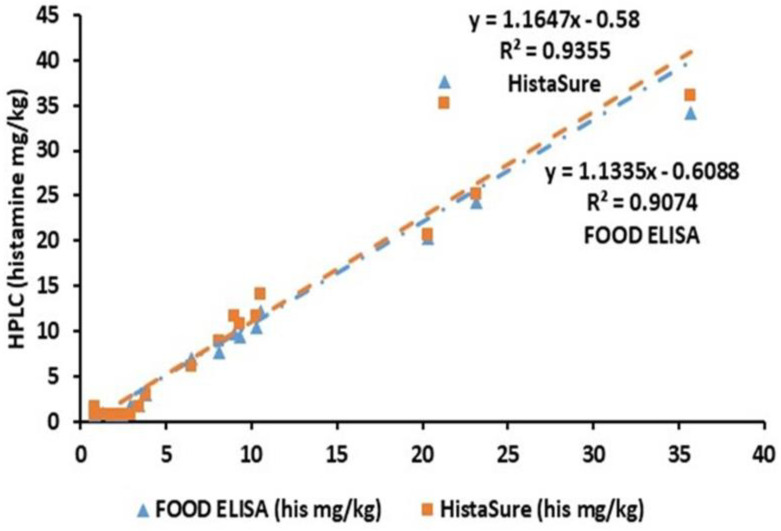
Correlation results of two quantitative test kits against the HPLC method in determining histamine in various fish products.

Figure 2 shows the correlation between the HPLC methods with two quantitative test kits in analyzing histamine in various fish products. Both test kits had a good correlation with HPLC although the HistaSure ELISA had slightly better correspondence. In our previous study with different test kits, a good correlation was also obtained with the HPLC method against histamine FOOD ELISA (previously named as FOOD EIA) (R^2^ = 0.9132) which closely supported our recent findings [23]. In the related study, about 91% of the results for the analyzed samples were found significantly similar to HPLC results indicating compatibility of the FOOD ELISA kit. However, in the current study, only 56.3% of the histamine values were significantly similar to each other (*p* > 0.05). One of the main differences can be attributed to the differences in the detection limits of both methods. In the previous study, the LOD value for HPLC was taken as the same as the FOOD EIA method as <2.5 mg/kg according to the test kit catalog and previous studies on HPLC. The LOD values with recent FOOD ELISA was improved by the company as <0.75 mg/kg for both test kits [39,40]. Similarly, the LOD values were reanalyzed for various traditional fish products and a lower value was obtained as <0.85 in our previous study [41]. In fact, 34.4% of the overall samples significantly differed due to the histamine values were found under detection limits, which are different for both methods. Therefore, by taking into account both values, total similarity adds up to 90.7% indicating the current study still supports the earlier findings [23]. Moreover, in the current study, different samples were also used adding up the differences in some samples due to the possible matrix effect.

The higher correlation was obtained between the results of the HPLC method and the HistaSure ELISA although lower amounts of samples (48.8%) were significantly similar to one to another (*p* > 0.05). Similarly, the majority of the differences corresponded to the values below detection limits (40.6%). In the previous study, lower correlation values were reported between the HPLC method, and Veratox and Histaquant test kits as R^2^ = 0.8793 and R^2^ = 0.7905, respectively [23].

**Figure 3:**
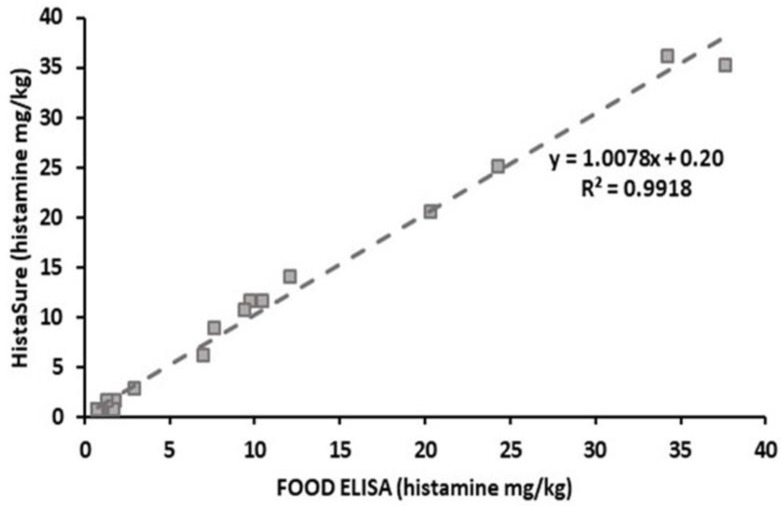
Correlation results of Food ELISA test kit and HistaSure ELISA (Fast Track) in determining histamine in various fish products.

Two quantitative test kits had the highest correlation with each other (R^2^ = 0.9918, Figure 3). Histamine values of 78.1% of the samples analyzed by both methods were significantly supported by each other (*p* > 0.05). The producer company also reported good recoveries, high sensitivity and reproducibility for both kits. However, only HistaSure ELISA had AOAC approval. Therefore, the Histamine FOOD ELISA kit has also a potential for application of AOAC certification.

Semi-quantitative test kits are very important for analyzing toxic compounds in food products. Such assays provide quick results for the food companies during monitoring of these compounds at the HACCP application of food processing. The results of both semi-quantitative test kits closely supported the quantitative values obtained by 4 quantitative methods. The results of 6 cut-off values of the old HistaSure (Histagold) kit disagreed with the quantitative methods, while only two cut-off values of HistaSure Lateral Flow test kit did not support the quantitative histamine results. However, both cut-off values (10 and 15 mg/kg) were found very close to the results of IC-MS/MS method as 9.03 and 11.25 mg/kg, respectively. A similar situation was also obtained with the Histagold test kit. However, negative values were also found with different replicates using this kit indicating the advantage of using more replicates for such kits. More cut-off values were applied for the Histagold test kit since the samples were tested both at the authors’ laboratory and the test kit producer company (LDN, Nordhorn, Germany). In the previous study [23], four semi-quantitative test kits were compared to the results of HPLC. The results showed that HistaSure by LDN (which is the oldest version of HistaSure using fluorescence end testing) and TRANSIA tube histamine kits are suitable for analyzing histamine in traditional fish products.

Testing histamine in fish and fish products is important for seafood processors in their HACCP program to address the hazard of scombrotoxin (histamine) formation. Histamine is a decomposition product of histidine and formed by decarboxylation activity during the growth of certain bacteria in some fish species. The amount of the amine that forms depends on bacterial species, the temperature and exposure time, and may exceed even over 1000 mg/kg [2,27]. Fish containing high histamine levels has been associated with many examples of HFPs which is a major health problem for consumers. Scombrotoxic fish often contains histamine values in over 200 mg/kg but such fish may be randomly dispersed within a lot. For large fish, histamine is usually found at variable levels even within individual fish. Quality control measures designed to minimize the number of HFP cases, require that the determination of histamine levels in fish products must be in the range of approximately 10 to 200 mg/kg. It was also reported that good quality fish contains less than 10 mg/kg histamine, while a level of 30 mg/kg indicates significant deterioration, and 50 mg/kg is considered evidence of definite decomposition. The defect action level, at which regulatory actions must be taken, for histamine is given as 50 mg/kg [20]. However, FDA guidelines suggest using 17 mg/kg histamine for HACCP plans to avoid the histamine health risk [9]. For an effective HACCP plan in the fish processing plant, rapid histamine testing is necessary for monitoring, since histamine and other BAs can continue to form even at a chilled temperature; therefore, the time to take action is very crucial [2,27]. Commercial test kits based on immunoassays are often used for such purposes either for quantitative or semi-quantitative analysis. The commercial test kits are usually obliged to be validated against a reference method in order to gain a buyer’s confidence. HistaSure ELISA (Fast Track) histamine test kit originally validated against Codex-approved fluorescence method and has AOAC certification, while the FOOD ELISA method was compared to the EC-approved HPLC method in our earlier study [23]. Since matrix effect and other drawbacks were reported for both reference (officially approved) methods [8,25,27,28,33], the recent comparison with IC-MS/MS analytical method without a derivatization step which avoids the effect of sample matrix provides valuable information on the performances of these quantitative test kits as well as the semi-quantitative methods used in this work.

Analysis of BAs other than histamine is also important in terms of food safety and quality. In the case of histamine toxicity, the potentiating effect of other BAs present in foods such as tyramine, putrescine and cadaverine was reported due to their competition with histamine-metabolizing enzymes [4,42,43]. Therefore, even low dietary intake of histamine can cause clinical signs of food intolerance [22,43]. Tyramine, tryptamine and phenylethylamine have been reported as vasoactive amines [2]. The main dietary intake of tyramine and other vasoactive amines comes from cheese and some fermented foods such as fish. Moreover, the toxicity of dietary tyramine and phenylethylamine is also reported. About 100–800 mg/kg tyramine and 30 mg/kg phenylethylamine have been reported to be toxic doses in foods [42]. Tyramine intoxication is commonly associated with cheeses. Tyramine is metabolized by monoamine oxidase. Inhibition of the activity of this enzyme by antidepressant drugs and ingestion of food high in tyramine can cause a hypertensive crisis [26]. The main dietary intake of tyramine and other vasoactive amines comes from cheese and some fermented foods. The accumulation of BAs in certain foods, particularly fermented fish is also linked to the formation of carcinogenic nitrosamines [2]. Several studies tried to link different levels of BAs with the spoilage of fish species [13,44,45]. Therefore, they obtained two different quality indexes depending on the levels of BAs in fish products.

**Figure 4:**
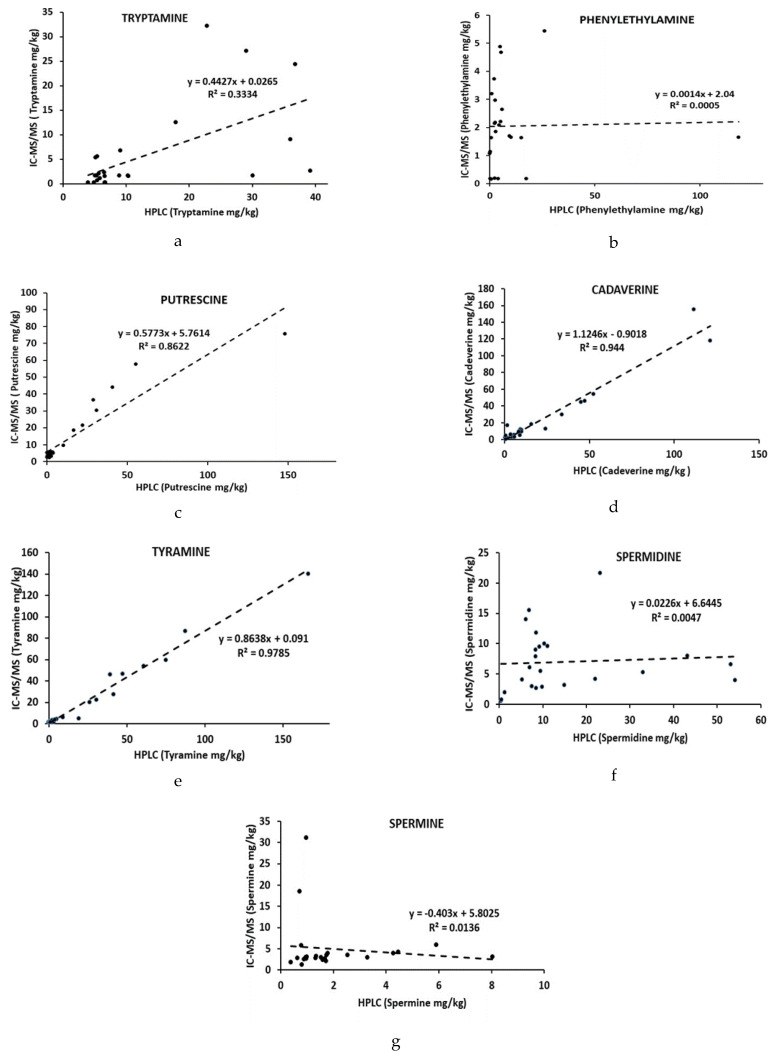
Correlation results between the IC-MS/MS and HPLC methods in determining seven biogenic amines in various fish products. ((**a**) Correlation results for Tryptamine; (**b**) Correlation results for Phenylethylamine; (**c**) Correlation results for Putrescine; (**d**) Correlation results for Cadeverine; (**e**) Correlation results for Tyramine; (**f**) Correlation results for Spermidine; (**g**) Correlation results for Spermine).

Table 2 represents the levels of 7 BAs analyzed by the IC-MS/MS and HPLC methods. A high correlation was observed between two methods in determining tyramine, cadaverine and putrescine as R^2^ = 0.9785, R^2^ = 0.944, and R^2^ = 0.8622, respectively (Figure 4c–e). However, worse correlations were obtained with other BAs (tryptamine, phenylethylamine, spermidine and spermine, Figure 4a,b,f,g). The main reasons may be attributed to the drawbacks previously reported for the HPLC method for determining BAs [26,27,33,41]. In our earlier study, lower recoveries were observed for these amines for several fish samples processed with different methods analyzed by HPLC. Low recoveries for all amines were particularly reported for marinated samples that might be affected by the low pH of the products [41]. In contrast, higher recoveries were obtained for histamine in different fish products by both HPLC and IC-MS/MS methods that confirming better correlation between two methods for histamine analysis [27,41]. Although some drawbacks were also observed for analysis of spermidine and spermine using the IC-MS/MS method, the LOD values were still higher than the values obtained by HPLC [26,27,41]. Statistical analysis did not confirm the correlation values since the results obtained for the most samples for BAs statistically differed from each other with some exceptions (*p* < 0.05).

It has been reported that traditional HPLC methods suffer from various drawbacks such as cumbersome sample preparation, problems of derivatization stability, by-products interference, complex instrumentation, and long analysis time. For ion chromatography without derivatization, as applied in the current study, offers a shorter analytical time and less interference with by-products [26,27,33]. Moreover, it is also a suitable method for marinated samples since ion chromatography was suggested being a more suitable approach for polar amines allowing a rather straightforward separation since at low pH all analytes exist in the ionized form [26,27]. Therefore, the IC-MS/MS method should be advised to be further investigated using inter-laboratory analysis for its suitability as a reference method for monitoring BAs in food products in terms of food safety and quality.

## 3. Materials and Methods

### 3.1. Chemicals

All chemicals and solvents used were of analytical and chromatographic grade provided from various companies (Fluka, Buchs, Switzerland; Sigma-Aldrich, St. Louis, MO, USA; Merck, Darmstadt, Germany; Carlo Erba, Milano, Italy; VWR Prolabo, Leuven, Belgium; Amresco, Cleveland, OH, USA). The water used for chromatographic separations and preparation of solutions was obtained from a Milli-Q water purification system (Millipore, Burlington, VT, USA) for IC-MS/MS analysis. For HPLC analysis, the water and acetonitrile were HPLC grade and water was obtained from VWR Prolabo (Leuven, Belgium).

### 3.2. Samples

Fish samples were mainly provided from markets/supermarkets of both Slovenia and Turkey either from local producers, supermarkets, or directly from the producer companies. Some products were originated from other countries (Croatia, Denmark, Germany, Italy, Lithuania, Norway, Romania, and Scotland). The products were transferred to the laboratory using cold storage conditions and kept in cold storage either at 4 °C or −18 °C when necessary. In total, 32 fish products were used for the study. Processing types of the products were brined (anchovy, Atlantic bonito), dry salted (herring), fermented (herring), marinated (anchovy, herring, salmon), smoked (anchovy, salmon, tuna), fish paste (anchovy, tuna, salmon), canned (sardine), and fresh sample (anchovy). There were also special types of products in combination with different processing techniques such as marinated herring with smoked salmon taste. The brined samples were in different variations and some being suspected as partially fermented. The first type of brined products was originated from Italy (bought in Slovenia) was stored in oil and the second type was called *Lakerda* (a Turkish and Greek-style salted fish) known as lightly preserved fish with varying salt content and also suspected as partly fermented. The third type originated from Italy was anchovy prepared in herbs called *Acciughette*, by the producer. The species of the fish used to produce the products were not given here due to uncertainty of species origin of some commercial fish products.

In order to comply with the cut-off values in the semi-quantitative test kits, some samples were selected from the previously analyzed fish products for BAs using the HPLC method [41,46] and then kept frozen after homogenization. However, for possible BA losses in these samples, they were re-analyzed using the HPLC method along with IC-MS/MS and 4 test kits.

### 3.3. Sample Preparation

All samples were brought to the laboratory in cold storage conditions. Each fish sample was homogenized in a food processor (Arçelik, K-1631 P Valso Plus, 2.2 L capacity, Eskişehir, Turkey) for 5 minutes and divided into three parts necessary for analysis using described tested procedures. Each homogeneous product was packed in a plastic bag and stored at −18 °C or cold storage, when necessary, prior to the analysis. Two distinctive extraction procedures were applied for the comparison study. The first one was used for IC-MS/MS and immunoassay procedures, the second one was used for the HPLC procedure. The sample transfer to the test kit producing company (LDN, Nordhorn, Germany) was carried out by a commercial cargo service using cold storage within a day. The samples were defrosted prior to analysis. Other sample transfers from authors’ laboratories either to/from Turkey to Slovenia were carried out in cold storage conditions by the authors within 10 h.

The extraction of BAs for IC-MS/MS and immunoassay analysis was done by weighing ten grams of homogeneous fish sample and adding 100 mL of Milli-Q water. The obtained mixture was homogenized at 25,000 rpm using Silent Crusher M (Heidolph, Schwabach, Germany) for about 1 min and left in the ultrasound bath for an additional 5 min. Afterward, the mixture was pre-filtered using Whatman paper (No. 1). The first 2–3 mL of filtrate was discarded. Prior to the analysis, the final filtration was done using a disposable syringe and Millex-LH filters (cat. No. SLLHRO4NL). Again the first 2–3 drops were discarded in order to condition the filters.

To extract BAs for HPLC analysis, 10 mL of 0.4 mol/L perchloric acid was added to 5 g of sample, and the mixture was homogenized by Ultra-Turrax Homogenizer (IKA T25, Digital, Staufen, Germany) in an ice bath and centrifuged (MPW 350 R. MPW Med. Instruments, Warsaw, Poland) at 2440 g at 4 °C for 10 min. The supernatant was collected and the residue was extracted again with 10 mL of 0.4 mol/L perchloric acid solution. Both supernatants were combined and filtered through Whatman paper (No. 42). The final volume was adjusted to 25 mL with 0.4 mol/L perchloric acid.

### 3.4. IC-MS/MS Procedure

Analysis of BAs using IC-MS/MMS was carried out according to Kočar et al. [27]. The method was performed on a Perkin Elmer HPLC (Shelton, CT, USA) consisted of a PE200 binary pump and PE200 autosampler. Cation exchange column IonPac (4 × 50 mm, Dionex, Sunnyvale, CA, USA) was used for the separation of BAs. Detection was performed with a 3200 QTrap mass spectrometer (Applied Biosystems, Foster City, CA, USA) in a multiple reaction monitoring mode (MRM). The ionization used was electrospray with the following ion source experimental conditions: ion spray voltage 5500 V, temperature 400 °C, curtain gas setting 20 psi, ion spray gas setting 20 psi and auxiliary gas setting 60 psi, respectively. The conditions for MRM monitoring (fragmentation conditions and fragment intensities) were optimized for each analyte separately to obtain the highest possible sensitivity [27].

### 3.5. HPLC Procedure and Derivatization Step

The HPLC procedure was carried out according to the procedures used in our earlier publications [23,41,46]. This method involves dansyl derivatization. HPLC equipment was Shimadzu Prominence LC-20 AT series (Tokyo, Japan) with autosampler (SIL20AC, Shimadzu, Tokyo, Japan), a Diode Array Detector (SPD-M20A, Shimadzu, Tokyo, Japan) and Intertsil column (GL Sciences, ODS-3, 5 μm, 4.6 × 250 mm).

### 3.6. ELISA Procedures

Four different ELISA test kits were used in this study. All test kits were made by the same producer and were developed for the fast non-expensive estimation of the histamine content in the fish products. The extraction of the histamine from the fish samples was the same as described for the IC-MS/MS procedure. Afterward, the sample handling was done as demanded by individual test kits. The analysis of test kits was carried out according to the instructions in the catalogs of the tests kits provided by the producer company [39]. The analyses of histamine in fish products were performed by the test kit producing company with the exception of HistaSure New Lateral Flow test kit was run at the authors’ laboratory. Some of the replicates relating to the Histagold test kit were also completed at the authors’ laboratory for confirmation.

Two of the investigated test kits were quantitative and two of them were semi-quantitative kits. The test kits are provided from Labor Diagnostika Nord (LDN), Germany. One of the test kits is called Histamine FOOD ELISA (Ref. No. FC-E 3100), previously known as Histamine FOOD EIA (Also known as RIDASCREEN, Art No. R1604 distributed by R-Biopharm, Inc., Marshall, MI, USA). It is a quantitative test kit with a total analytical time is less than 2 h. The assay principle is competitive ELISA. The second quantitative kit was HistaSure ELISA, Fast Track, which was AOAC Approved test kit (Certificate No. 021402 and Ref. No. FC-E 3600). This test kit is also a competitive ELISA with a total assay time of around 35–40 min. In both methods, the extraction solution is water.

Two semi-quantitative test kits were used in this study, namely Histagold which is a modified version of HistaSure Fish (Catalogue No. FC L-3300) and HistaSure Fish Rapid Test (Lateral Flow assay) (Catalogue No. FC-L 3200). These test kits are similar to each other with differences arising from the end testing technique. The company firstly produced HistaSure Fish which uses a fluorescence dye to label the antibody. Total assay time including sample preparation takes between 20–30 min. The method is set for 50 mg/kg histamine value. However, by adjusting the extract volume with different dilution approaches (from 5 to 200 mg/kg), lower or higher amounts of histamine set value can be arranged with the method. The extraction solution is water. The second semi-quantitative test kit is a modified version of the original HistaSure fish by the LDN Company. Both Histagold and HistaSure fish kits had lots of similarities using the same antiserum. Therefore, only Histagold kit was used in this study. However, the kit was withdrawn from the market after the study. In the Histagold kit, a certain amount of diluted acylated buffer is added directly to the sample pad which is called ‘LF cassette’ and incubated for 5 min, then the results as one or two red bands are read visually under normal light within ten minutes. One band is ‘positive’ which means the value is above the relating cut-off value, two bands represent ‘negative’ meaning the value is below the relating cut-off value. HistaSure Lateral Flow assay is a modified version of Histagold. This test kit is currently on the market. The principle of the kit is; after a simple and quick water extraction step, the histamine in the sample is quantitatively derivatized into *N*-acylhistamine. After dilution of the *N*-acylated histamine in running buffer, the Lateral flow device is added to the sample. The amount of immunogold labeled antibody bound to the solid phase histamine is inversely proportional to the histamine concentration in the sample. The modification is the end reading system which does not use an LF cassette but uses a lateral flow device. Total assay time is close to the previous kit.

More information can be obtained by the test kits’ websites or by contacting directly to each company. Test kit applications to samples were carried out according to the instruction manual of each kit [39]. Equipment and some materials that are not provided with test kits were ELISA microplate reader (Dynex Electr., Opsys MR, Chantilly, VA, USA), microplate shaker (Heidolph, Rotamax 120, Schwabach, Germany). Automatic pipettes with varying measuring capacity were used by different models and companies, including Gilson Pipetman Concept (Villers le Bel, France); HTL Labmate plus (Warsaw, Poland), Brand (Handystep, Wertheim, Germany), Eppendorf (Hamburg, Germany).

Semi-quantitative calculations were performed according to the manufactures’ instructions which were carried out by the technical service of the test kit producer company (LDN, Germany). If the standard curve of each trial relating to test kit was not approved by the company’s technical service or accepted as poor, the results were not included in the evaluation and such sample was indicated as ‘Not analyzed’ in the relating tables.

Analyses of histamine using semi-quantitative test kits were carried out 3 times by different applicators. Repetition was usually carried out close to actual quantitative results obtained pre-analysis either by HPLC or IC-MS/MS. More than one cut-off value was applied for various samples. If the actual value was identified before the semi-quantitative analysis, fewer cut-offs were used. If not, then a wide range of cut-off values was tried.

### 3.7. Statistical Analysis

The data obtained were analyzed using analysis of variance (ANOVA) and when significant differences were found, comparisons among means were carried out by using Tukey test (*p* < 0.05) by JMP 5.0.1 (SAS Institute, Inc., Cary, NC, USA), Sokal and Rohlf [47]. Linear regression analysis was calculated from Microsoft EXCEL, 2003.

## 4. Conclusions

In conclusion, the methods used in this study had a good correlation in determining histamine in different fish and fish products processed by various methods and therefore, they can be used for monitoring histamine in the seafood industry. However, a poor correlation was observed between HPLC and IC-MS/MS method in estimating other BAs. Therefore, further studies are required to confirm the possible effects. This study provides a comparative evaluation of several methods in terms of their suitability in determining biogenic amines in fish products for both monitoring and regulatory purposes.

## Figures and Tables

**Table 1:** Histamine values (mg/kg) of various fish products analyzed by 4 quantitative (IC-MS/MS, HPLC, Food ELISA and HistaSure ELISA Fast Track) methods and 2 semi-quantitative test kits.

		Quantitative Methods				Semi-Quantitative Methods			
		(Histamine mg/kg)				New HistaSure-Lateral Flow		Old HistaSure–HISTAGOLD	
No.	Samples	IC-MS/MS	HPLC	FOOD ELISA	HistaSure ELISA, Fast Track	R	Cut-Off Values	R	Cut-Off Values
1	Anchovy, Fresh-1	4.51 ± 0.67 ^a^	3.45 ± 0.02 ^b^	1.81 ± 0.80 ^c^	1.61 ± 0.20 ^c^	N	10 mg/kg	N	**5**, 25, 50 mg/kg
2	Anchovy, Fresh-2	1.98 ± 0.21 ^a^	1.97 ± 0.42 ^a^	<0.75 ^b^	<0.75 ^b^	N	10 mg/kg	N	5, 10, 50 mg/kg
3	Anchovy, Fresh-3	NA	2.14 ± 0.09 ^a^	<0.75 ^b^	<0.75 ^b^	N	10 mg/kg	N	5, 10, 50 mg/kg
4	Anchovy, Brined-1 (15% brine, lab. made)	2.59 ± 0.98 ^a^	2.42 ± 0.03 ^a^	<0.75 ^b^	<0.75 ^b^	N	10 mg/kg	N	5, 50 mg/kg
5	Anchovy, Brined-2 (15% brine), Turkey	NA	319.05 ± 0.72 ^a^	320.00 ± 5.65 ^a^	>250 ^a^	P	50, 100, 200 mg/kg	P	50, 200 mg/kg
				(>250)					
6	Anchovy, Brined-3, Turkey	15.17 ± 0.40 ^a^	10.50 ± 0.18 ^b^	12.08 ± 1.25 ^b^	14.11 ± 0.18 ^a^	P	10 mg/kg	P	10, 15 mg/kg
						N	25 mg/kg	N	15, 25, 50 mg/kg
7	Anchovy, Brined-5, Turkey	36.20 ± 2.56 ^a^	35.71 ± 0.86 ^a^	34.19 ± 2.79 ^a^	36.10 ± 1.50 ^a^	P	25 mg/kg	P	10, 25 mg/kg
						N	50, 100 mg/kg	N	50, 100 mg/kg
8	Anchovy, Brined-5 (20% brine), Turkey	NA	72.94 ± 1.86 ^a^	83.30 ± 7.92 ^a^	73.38 ± 2.74 ^a^	P	50 mg/kg	P	50 mg/kg
						N	100 mg/kg	N	100 mg/kg
9	Anchovy, in herbal sauce, Italy	11.10 ± 0.01 ^a^	10.33 ± 0.55 ^a^	10.42 ± 0.68 ^a^	11.60 ± 0.20 ^a^	P	NA	P	5, 10 mg/kg
						N	NA	N	15, 25 mg/kg
10	Anchovy, Dry-salted in oil, Croatia	6.06 ± 0.63 ^a^	6.47 ± 0.31 ^a^	6.95 ± 0.91 ^a^	6.15 ± 0.20 ^a^	N	10, 15 mg/kg	P	5 mg/kg
								N	5, 10, 15, 50 mg/kg
11	Anchovy, Marinated-1 (in oil & vinegar), Italy	NA	2.02 ± 0.05 ^a^	2.10 ± 0.36 ^a^	1.50 ± 0.20 ^a^	N	10 mg/kg	N	5, 50 mg/kg
12	Anchovy, Marinated-2, Turkey	NA	3.49 ± 0.17 ^a^	3.88 ± 0.50 ^a^	2.70 ± 0.26 ^b^	N	10 mg/kg	P	**5 mg/kg**
								N	10, 50 mg/kg
13	Anchovy, Paste, Italy	3.06 ± 0.03 ^a^	3.82 ± 0.11 ^b^	2.90 ± 0.18 ^a^	2.90 ± 0.20 ^a^	N	10 mg/kg	N	5, 10, 50 mg/kg
14	Anchovy-Smoked & Marinated, Turkey	2.36 ± 0.48 ^a^	1.85 ± 0.07 ^a^	<0.75 ^c^	<0.75 ^c^	N	10 mg/kg	N	5, 10, 50 mg/kg
15	A. Bonito, Lakerda-1, Turkey	1.78 ± 0.06 ^a^	<0.85 ^b^	<0.75 ^c^	<0.75 ^c^	N	10 mg/kg	N	5, 10, 50 mg/kg
16	A. Bonito, Lakerda-2, Turkey	2.59 ± 0.50 ^a^	1.07 ± 0.06 ^b^	1.00 ± 0.34 ^b^	<0.75 ^c^	N	10 mg/kg	N	5, 10 mg/kg
17	A. Bonito, Lakerda-3, Turkey	20.47 ± 0.45 ^a^	20.29 ± 0.70 ^a^	20.34 ± 1.34 ^a^	20.63 ± 1.10 ^a^	P	5, 10, 15 mg/kg	P	15 mg/kg
						N	50, 200 mg/kg	N	50, 200 mg/kg
18	A. Bonito, Lakerda-4, Turkey	196.67 ± 0.58 ^a^	205.87 ± 3.15 ^a^	158.2 ± 6.36 ^b^	201.59 ± 12.18 ^a^	P	200 mg/kg	P	50, 200 mg/kg
19	Herring, Dry salted-1, Turkey	2.36 ± 0.58 ^a^	2.91 ± 0.11 ^a^	1.74 ± 0.98 ^ab^	<0.75 ^c^	N	10 mg/kg	N	5, 10, 50 mg/kg
20	Herring, Dry salted-2, Turkey	9.03 ± 0.20 ^a^	8.14 ± 0.46 ^a^	7.60 ± 0.16 ^a^	8.91 ± 0.20 ^a^	P N	**10 mg/kg**	P N	5, **10 mg/kg**
							15 mg/kg		10, 15, 25 mg/kg
21	Herring, Marinated-1, Romania	1.59 ± 0.12 ^a^	1.28 ± 0.02 ^b^	<0.75 ^c^	<0.75 ^c^	N	10 mg/kg	N	5, 10, 50 mg/kg
22	Herring, Marinated-2, Turkey	1.38 ± 0.54 ^a^	<0.85 ^b^	<0.75 ^c^	<0.75 ^c^	N	10 mg/kg	N	5, 10, 50 mg/kg
23	Herring, Fermented-1, Denmark	11.25 ± 0.49 ^a^	8.98 ± 0.36 ^b^	9.73 ± 1.39 ^ab^	11.60 ± 0.40 ^a^	P N	10, **15 mg/kg**	P N	10, **15 mg/kg**
							25, 50 mg/kg		15, 25, 50 mg/kg
24	Herring, Fermented-2, Denmark	12.55 ± 3.10 ^a^	9.31 ± 0.40 ^a^	9.42 ± 1.09 ^a^	10.70 ± 0.40 ^a^	N	25, 50 mg/kg	P N	5, 10 mg/kg
									15, 25, 50 mg/kg
25	Salmon, Smoked, Norway	1.67 ± 0.09 ^a^	1.81 ± 0.06 ^a^	<0.75 ^b^	<0.75 ^c^	N	10 mg/kg	N	5, 10, 50 mg/kg
26	Salmon, Gravadlax-1, Scotland	21.99 ± 1.97 ^a^	23.12 ± 1.23 ^a^	24.30 ± 4.38 ^a^	25.10 ± 1.80 ^ab^	P N	15 mg/kg	P N	10, 15 mg/kg
							25, 50, 100 mg/kg		25, 50, 100 mg/kg
27	Salmon, Gravadlax-2, Scotland	35.10 ± 5.80 ^a^	21.29 ± 4.00 ^b^	37.60 ± 2.83 ^a^	35.20 ± 2.43 ^a^	P N	10, 15 mg/kg	P N	15, 25 mg/kg
							25, 50, 100 mg/kg		50, 100 mg/kg
28	Salmon paste, Germany	NA	4.95 ± 0.07 ^a^	3.58 ± 0.13 ^b^	2.70 ± 0.10 ^b^	N	10 mg/kg	P N	5 mg/kg
									10, 50 mg/kg
29	Sardine, Canned, Slovenia	NA	<0.85 ^a^	<0.75 ^a^	<0.75 ^a^	N	10 mg/kg	N	5, 10, 50 mg/kg
30	Tuna, Paste, Denmark	2.10 ± 0.40 ^a^	<0.85 ^b^	1.30 ± 0.13 ^c^	1.60 ± 0.10 ^a^	N	10 mg/kg	N	5, 10, 50 mg/kg
31	Tuna, Smoked, Lithuania	0.30 ± 0.02 ^a^	<0.85 ^a^	<0.75 ^a^	<0.75 ^a^	N	10 mg/kg	N	5, 10, 50 mg/kg
32	Tuna, Smoked, Europe	2.60 ± 0.01 ^a^	<0.85 ^b^	<0.75 ^c^	<0.75 ^c^	N	10 mg/kg	P	**5 mg/kg**
								N	10, 50 mg/kg

Different superscript lowercase letters (a, b, c) in the same row represent statistical differences amongst the methods (*p* < 0.05). NA: Not analyzed, R: Results, P: Positive (means the value is above this cut-off level), N: Negative (means the value is below this cut-off level). Histamine LOD values for <0.85 mg/kg HPLC, <0.75 mg/kg Food ELISA and HistaSure ELISA, *Gravadlax*: Marinated and fermented. The bold cut-off values represent the disagreement of the method with at least one of the quantitative method.

**Table 2:** Comparison of the IC-MS/MS and HPLC methods for analyzing 7 biogenic amines in fish products.

		Biogenic Amines (mg/kg)						
Samples	Methods	TRP	PHE	PUT	CAD	TYR	SPD	SPM
Fresh Anchovy-1, Turkey	IC-MS/MS	1.63 ± 0.07 ^a^	1.63 ± 0.01 ^a^	4.43 ± 0.34 ^a^	12.15 ± 0.49 ^a^	3.40 ± 0.09 ^a^	0.53 ± 0.10 ^a^	2.62 ± 0.75 ^a^
	HPLC	8.90 ± 0.96 ^b^	0.84 ± 0.18 *^2,b^	2.16 ± 0.15 ^b^	9.36 ± 0.59 ^b^	3.10 ± 0.04 ^a^	0.36 ± 0.03 *^6,b^	1.69 ± 0.09 ^b^
Fresh Anchovy-2, Turkey	IC-MS/MS	1.57 ± 0.04 ^a^	0.18 ± 0.03 *^1,a^	3.21 ± 0.25 ^a^	5.38 ± 0.01 ^a^	1.44 ± 0.14 ^a^	0.73 ± 0.21 ^a^	2.33 ± 0.87 ^a^
	HPLC	10.34 ± 0.11 ^b^	0.28 ± 0.04 *^2,a^	1.68 ± 0.20 ^b^	5.52 ± 0.51 ^a^	<0.90 ^b^	0.47 ± 0.04 *^6,b^	1.59 ± 0.02 ^a^
Anchovy, Brined-1 (15% brine, lab. made)	IC-MS/MS	2.67 ± 1.03 ^a^	0.19 ± 0.01 *^1,a^	5.39 ± 1.84 ^a^	11.35 ± 0.92 ^a^	2.16 ± 0.06 ^a^	2.85 ± 0.21 ^a^	1.75 ± 0.35 ^a^
	HPLC	39.18 ± 2.06 ^b^	2.40 ± 0.16 ^b^	0.32 ± 0.07 *^3,b^	10.20 ± 0.31 ^a^	1.56 ± 0.17 ^b^	9.90 ± 0.34 ^b^	0.39 ± 0.06 *^7,b^
Anchovy, Brined-2, Turkey	IC-MS/MS	24.40 ± 0.70 ^a^	3.73 ± 0.03 ^a^	43.95 ± 1.90 ^a^	3.40 ± 0.88 ^a^	46.02 ± 0.90 ^a^	5.46 ± 3.17 ^a^	5.83 ± 1.70 ^a^
	HPLC	36.85 ± 2.90 ^b^	2.25 ± 1.42 ^a^	40.81 ± 0.59 ^a^	4.57 ± 0.38 ^a^	39.34 ± 0.74 ^b^	9.48 ± 0.57 ^b^	0.79 ± 0.05 ^b^
Anchovy, Brined-3, Turkey	IC-MS/MS	32.23 ± 0.93 ^a^	4.67 ± 0.21 ^a^	57.57 ± 1.19 ^a^	3.05 ± 0.77 ^a^	53.98 ± 0.69 ^a^	7.93 ± 0.69 ^a^	2.99 ± 0.84 ^a^
	HPLC	22.84 ± 0.83 ^b^	5.51 ± 0.60 ^a^	55.36 ± 1.35 ^a^	3.84 ± 0.36 ^a^	60.90 ± 0.20 ^b^	8.31 ± 0.33 ^a^	0.95 ± 0.09 ^b^
Anchovy, in herbal sauce, Italy	IC-MS/MS	1.69 ± 0.06 ^a^	2.17 ± 0.07 ^a^	3.57 ± 0.09 ^a^	5.12 ± 0.24 ^a^	2.40 ± 0.07 ^a^	4.09 ± 0.71 ^a^	3.26 ± 0.09 ^a^
	HPLC	10.32 ± 0.18 ^b^	2.81 ± 0.14 ^b^	1.36 ± 0.16 ^b^	9.15 ± 1.12 ^b^	2.30 ± 0.20 ^a^	5.21 ± 1.10 ^a^	1.35 ± 0.17 ^b^
Anchovy, Dry-salted in oil, Croatia	IC-MS/MS	1.55 ± 0.01 ^a^	2.08 ± 0.10 ^a^	5.46 ± 0.46 ^a^	9.49 ± 0.62 ^a^	3.99 ± 0.31 ^a^	9.48 ± 0.09 ^a^	2.87 ± 0.75 ^a^
	HPLC	6.58 ± 0.16 ^b^	4.60 ± 0.08 ^b^	2.72 ± 0.20 ^b^	9.98 ± 0.34 ^a^	4.87 ± 0.30 ^b^	9.28 ± 0.14 ^a^	1.54 ± 0.13 ^b^
Anchovy, Paste, Italy	IC-MS/MS	5.56 ± 0.34 ^a^	2.21 ± 0.09 ^a^	4.97 ± 0.20 ^a^	12.50 ± 0.26 ^a^	6.18 ± 0.24 ^a^	11.81 ± 1.88 ^a^	3.56 ± 0.23 ^a^
	HPLC	5.42 ± 0.30 ^a^	5.32 ± 0.44 ^b^	4.12 ± 0.12 ^b^	24.20 ± 0.88 ^b^	8.98 ± 0.34 ^b^	8.46 ± 0.68 ^b^	2.54 ± 0.48 ^b^
Anchovy-Smoked & Marinated, Turkey	IC-MS/MS	1.62 ± 0.08 ^a^	1.64 ± 0.02 ^a^	3.30 ± 0.48 ^a^	3.19 ± 0.33 ^a^	1.33 ± 0.05 ^a^	8.98 ± 2.02 ^a^	2.82 ± 0.71 ^a^
	HPLC	5.34 ± 0.19 ^b^	10.31 ± 0.64 ^b^	2.74 ± 0.15 ^a^	6.07 ± 0.93 ^b^	0.66 ± 0.11 *^5,b^	8.34 ± 0.22 ^a^	0.63 ± 0.67 *^7,b^
A. Bonito, Lakerda-1, Turkey	IC-MS/MS	5.33 ± 0.40 ^a^	3.20 ± 0.32 ^a^	30.20 ± 0.98 ^a^	53.87 ± 6.29 ^a^	46.73 ± 1.97 ^a^	21.65 ± 2.28 ^a^	18.45 ± 3.14 ^a^
	HPLC	5.12 ± 0.28 ^a^	1.08 ± 0.14 *^1,b^	31.05 ± 1.14 ^a^	52.38 ± 3.54 ^a^	47.17 ± 2.46 ^a^	23.22 ± 1.52 ^a^	<0.71 ^b^
A. Bonito, Lakerda-2, Turkey	IC-MS/MS	0.19 ± 0.01 *^1,a^	1.63 ± 0.03 ^a^	2.80 ± 0.29 ^a^	2.59 ± 0.01 ^a^	1.38 ± 0.06 ^a^	3.99 ± 0.16 ^a^	2.94 ± 0.17 ^a^
	HPLC	4.88 ± 0.26 ^b^	15.16 ± 0.34 ^b^	<0.56 ^b^	1.53 ± 0.20 ^b^	3.02 ± 0.18 ^b^	54.16 ± 3.12 ^b^	3.30 ± 0.22 ^a^
A. Bonito, Lakerda-3, Turkey	IC-MS/MS	1.66 ± 0.07 ^a^	1.07 ± 0.84 ^a^	36.47 ± 0.93 ^a^	155.00 ± 1.41 ^a^	86.40 ± 3.89 ^a^	4.14 ± 1.17 ^a^	2.60 ± 0.95 ^a^
	HPLC	5.11 ± 0.04 ^b^	0.07 ± 0.07 *^2,b^	28.99 ± 0.79 ^b^	111.59 ± 2.37 ^b^	87.38 ± 2.48 ^a^	22.02 ± 0.69 ^b^	0.96 ± 0.12 ^b^
A. Bonito, Lakerda-4, Turkey	IC-MS/MS	2.11 ± 0.77 ^a^	0.18 ± 0.02 ^a^	3.39 ± 1.31 ^a^	9.60 ± 0.55 ^a^	2.03 ± 0.32 ^a^	3.15 ± 0.45 ^a^	3.04 ± 0.57 ^a^
	HPLC	5.68 ± 0.09 ^b^	17.49 ± 0.99 ^b^	0.43 ± 0.11 *^3,b^	8.46 ± 0.19 ^a^	0.62 ± 0.18 *^5,b^	15.01 ± 0.87 ^b^	0.99 ± 0.13 ^b^
Herring, Dry salted-1, Turkey	IC-MS/MS	0.18 ± 0.03 *^1,a^	0.18 ± 0.03 *^1,a^	5.53 ± 0.20 ^a^	18.37 ± 0.50 ^a^	1.52 ± 0.04 ^a^	9.61 ± 0.32 ^a^	2.78 ± 0.71 ^a^
	HPLC	6.73 ± 0.23 ^b^	4.11 ± 0.35 ^b^	2.98 ± 0.30 ^b^	15.95 ± 1.12 ^a^	0.45 ± 0.16 *^5,b^	11.10 ± 0.25 ^b^	1.33 ± 0.15 ^b^
Herring, Marinated-1, Romania	IC-MS/MS	27.13 ± 1.59 ^a^	1.85 ± 0.10 ^a^	2.66 ± 0.09 ^a^	3.61 ± 0.06 ^a^	27.28 ± 0.76 ^a^	2.67 ± 0.15 ^a^	2.50 ± 0.98 ^a^
	HPLC	29.06 ± 1.26 ^a^	2.84 ± 0.16 ^b^	0.32 ± 0.11 *^3,b^	4.38 ± 0.68 ^b^	41.56 ± 3.12 ^b^	8.44 ± 0.52 ^b^	0.89 ± 0.10 *^7,b^
Herring, Marinated-2, Turkey	IC-MS/MS	9.03 ± 0.35 ^a^	4.87 ± 0.23 ^a^	5.50 ± 0.70 ^a^	16.60 ± 1.56 ^a^	3.20 ± 0.08 ^a^	10.04 ± 0.66 ^a^	3.46 ± 0.64 ^a^
	HPLC	36.06 ± 1.52 ^b^	5.05 ± 0.10 ^a^	0.99 ± 0.33 ^b^	1.75 ± 0.10 ^b^	1.90 ± 0.16 ^b^	10.33 ± 0.78 ^a^	1.75 ± 0.02 ^b^
Herring, Fermented-1, Denmark	IC-MS/MS	6.77 ± 0.61 ^a^	2.97 ± 0.11 ^a^	18.55 ± 0.64 ^a^	44.37 ± 1.98 ^a^	22.23 ± 0.90 ^a^	15.50 ± 1.13 ^a^	3.89 ± 0.61 ^a^
	HPLC	9.12 ± 0.90 ^b^	2.72 ± 0.40 ^a^	16.88 ± 1.32 ^a^	45.08 ± 2.18 ^a^	30.42 ± 2.37 ^b^	6.89 ± 0.78 ^b^	1.78 ± 0.21 ^b^
Herring, Fermented-2, Denmark	IC-MS/MS	1.67 ± 0.02 ^a^	2.15 ± 0.02 ^a^	21.53 ± 0.85 ^a^	46.20 ± 3.14 ^a^	20.33 ± 0.12 ^a^	14.07 ± 0.51 ^a^	2.13 ± 0.35 ^a^
	HPLC	30.08 ± 3.12 ^b^	2.56 ± 0.30 ^a^	22.36 ± 2.08 ^a^	47.32 ± 2.08 ^a^	26.12 ± 2.08 ^b^	6.12 ± 0.42 ^a^	1.72 ± 0.30 ^b^
Salmon, Smoked, Norway	IC-MS/MS	12.53 ± 0.84 ^a^	5.43 ± 0.19 ^a^	75.60 ± 0.71 ^a^	118.15 ± 1.63 ^a^	140.25 ± 2.99 ^a^	3.00 ± 0.19 ^a^	3.88 ± 0.69 ^a^
	HPLC	17.88 ± 1.42 ^b^	26.18 ± 2.08 ^b^	148.08 ± 5.10 ^b^	121.18 ± 2.68 ^a^	166.38 ± 5.24 ^b^	7.44 ± 0.72 ^b^	4.28 ± 0.54 ^a^
Salmon, *Gravadlax*, Scotland	IC-MS/MS	0.66 ± 0.80 ^a^	2.64 ± 0.71 ^a^	9.71 ± 0.70 ^a^	29.59 ± 0.55 ^a^	59.70 ± 0.71 ^a^	6.03 ± 0.97 ^a^	3.13 ± 0.10 ^a^
	HPLC	5.38 ± 0.22 ^b^	5.92 ± 0.48 ^b^	10.48 ± 1.02 ^a^	33.88 ± 3.87 ^a^	75.12 ± 3.06 ^b^	6.98 ± 0.52 ^a^	8.03 ± 0.60 ^b^
Tuna, Paste, Denmark,	IC-MS/MS	0.18 ± 0.03 *^1,a^	1.70 ± 0.04 ^a^	5.53 ± 0.75 ^a^	5.89 ± 0.62 ^a^	3.59 ± 0.11 ^a^	8.02 ± 0.25 ^a^	4.20 ± 0.73 ^a^
	HPLC	6.62 ± 0.72 ^b^	9.44 ± 0.58 ^b^	3.74 ± 0.22 ^b^	3.86 ± 0.52 ^b^	3.72 ± 0.40 ^a^	43.22 ± 3.34 ^b^	4.46 ± 0.72 ^a^
Tuna, Smoked, Lithuania	IC-MS/MS	2.33 ± 0.55 ^a^	1.64 ± 0.01 ^a^	3.58 ± 0.35 ^a^	4.39 ± 0.36 ^a^	4.69 ± 0.08 ^a^	6.62 ± 0.52 ^a^	5.86 ± 0.21 ^a^
	HPLC	6.54 ± 0.34 ^b^	118.36 ± 6.12 ^b^	1.32 ± 0.26 ^b^	<0.87 ^*4, b^	19.06 ± 2.42 ^b^	53.12 ± 4.17 ^b^	5.90 ± 0.68 ^a^
Tuna, Smoked, Europe	IC-MS/MS	0.19 ± 0.01 *^1,a^	0.15 ± 0.00 *^1,a^	3.47 ± 0.57 ^a^	0.80 ± 0.07 ^a^	1.49 ± 0.12 ^a^	2.00 ± 0.00 ^a^	31.20 ± 0.57 ^a^
	HPLC	3.94 ± 0.18 ^b^	<1.50 ^a^	<0.56 ^b^	<0.66 ^b^	<0.87 ^b^	1.22 ± 0.24 ^b^	0.96 ± 0.20 ^b^

Different superscript lowercase letters (a, b) in the same column represent statistical differences amongst the methods (*p* < 0.05). Gravadlax: Marinated and fermented, TRP: Tryptamine, PHE: Phenylethylamine, PUT: Putrescine, CAD: Cadaverine, TYR: Tyramine, SPD: Spermidine, SPM: Spermine, HPLC: High Performance Liquid Chromatography, IC-MS/MS: Ion Chromatography Mass Spectrometry. Detection Limits: *1: <0.20, *2: <1.50, *3: <0.56, *4: <0.66, *5: <0.87, *6: <0.65, *7: <0.71.

## Data Availability

Not applicable.
